# A Novel Method for Analysing the Curvature of the Anterior Lens: Multi-Radial Scheimpflug Imaging and Custom Conic Fitting Algorithm

**DOI:** 10.3390/jimaging11080257

**Published:** 2025-08-01

**Authors:** María Arcas-Carbonell, Elvira Orduna-Hospital, María Mechó-García, Guisela Fernández-Espinosa, Ana Sanchez-Cano

**Affiliations:** 1 Departamento de Física Aplicada, Universidad de Zaragoza, 50009 Zaragoza, Spain; marcas@unizar.es (M.A.-C.); guisela.fernandez3@gmail.com (G.F.-E.); 2 Instituto de Investigación Sanitaria de Aragón (IIS Aragón), 50009 Zaragoza, Spain; 3 Instituto de Nanociencia y Materiales de Aragón (INMA), CSIC-Universidad de Zaragoza, 50009 Zaragoza, Spain; mmechogarcia@unizar.es

**Keywords:** lens curvature analysis, anterior chamber image segmentation, Scheimpflug imaging, age-related accommodation

## Abstract

This study describes and validates a novel method for assessing anterior crystalline lens curvature along vertical and horizontal meridians using radial measurements derived from Scheimpflug imaging. The aim was to evaluate whether pupil diameter (PD), anterior lens curvature, and anterior chamber depth (ACD) change during accommodation and whether these changes are age-dependent. A cross-sectional study was conducted on 104 right eyes from healthy participants aged 21–62 years. Sixteen radial images per eye were acquired using the Galilei Dual Scheimpflug Placido Disk Topographer under four accommodative demands (0, 1, 3, and 5 dioptres (D)). Custom software analysed lens curvature by calculating eccentricity in both meridians. Participants were analysed as a total group and by age subgroups. Accommodative amplitude and monocular accommodative facility were inversely correlated with age. Both PD and ACD significantly decreased with higher accommodative demands and age. Relative eccentricity decreased under accommodation, indicating increased lens curvature, especially in younger participants. Significant curvature changes were detected in the horizontal meridian only, although no statistically significant differences between meridians were found overall. The vertical meridian showed slightly higher eccentricity values, suggesting that it remained less curved. By enabling detailed, meridionally stratified in vivo assessment of anterior lens curvature, this novel method provides a valuable non-invasive approach for characterizing age-related biomechanical changes during accommodation. The resulting insights enhance our understanding of presbyopia progression, particularly regarding the spatial remodelling of the anterior lens surface.

## 1. Introduction

Studying the ocular structures involved in the accommodation process across different age groups can provide key insights into their behaviour, morphological variations, and the mechanisms underlying the progressive decline in accommodative ability.

Accommodation is a dynamic process that adjusts the eye’s optical power to maintain a clear retinal image as the viewing distance changes, particularly when shifting from far vision (FV) to near vision (NV) [1,2]. This process is mainly mediated by the crystalline lens, which alters its shape and curvature through the action of the ciliary muscle and zonular fibres. During NV tasks, the ciliary muscle contracts, reducing the tension on the zonules and allowing the lens to adopt a more convex shape, thereby increasing its refractive power. This structural and functional interaction is complemented by other physiological responses, such as those of the iris, which undergoes miosis, and the extraocular muscles, which contribute to ocular convergence, together forming the so-called accommodative triad [3]. The crystalline lens plays a pivotal role, and its biomechanical and anatomical integrity is essential for efficient accommodation. Over time, structural age-related changes in the lens lead to a reduction in accommodative amplitude (AA), ultimately leading to presbyopia [4,5].

Corneal topography has traditionally been used to analyse the curvature of the anterior corneal surface, especially through Placido disk-based systems. However, technological advances have led to the development of more sophisticated devices that combine topographic and tomographic data, allowing for a more comprehensive characterization of ocular structures, such as the one used in the present study. The selected tomographer integrates corneal curvature data obtained through Placido rings with elevation data generated by a dual Scheimpflug camera system. To better understand the anatomical changes involved in accommodation, advanced imaging technologies have become essential. While traditional corneal topography has been widely used to assess anterior corneal curvature, newer devices now integrate both topographic and tomographic data for a more complete evaluation of the anterior segment. In this study, we employed a tomographer that combines curvature data from Placido rings with elevation data captured by a dual Scheimpflug camera system [6]. This technology provides high-resolution, three-dimensional images of the anterior segment, including cross-sectional views of the cornea and the crystalline lens. It also enables the generation of pachymetry maps, anterior segment biometric data, and measurements of total corneal refractive power [7]. Due to their precision and reliability, rotating Scheimpflug systems are widely used in both clinical and research settings to evaluate the structures involved in accommodation [8].

In addition to the crystalline lens and other structures directly involved in the accommodative process, the anterior chamber depth (ACD) and anterior chamber volume (ACV) also play a crucial role in maintaining intraocular pressure, ocular alignment, and accommodation dynamics, given their impact on lens positioning and aqueous humour flow [9]. Alterations in their anatomy or in the dynamics of the aqueous humour can lead to clinically relevant pathologies such as glaucoma, highlighting the importance of their precise measurement [10,11]. Moreover, changes in ACD can be influenced not only by age-related physiological changes but also by biomechanical adaptations associated with the accommodative effort required to maintain retinal focus in NV [12].

Recent advances in ocular imaging technology have significantly enhanced our ability to analyse fine anatomical structures in vivo with precision and reliability. High-resolution systems such as Scheimpflug imaging, anterior segment optical coherence tomography (OCT) [13,14], and swept-source OCT (SS-OCT) [15,16,17] have expanded the scope of anterior segment evaluation, enabling detailed visualization of dynamic ocular processes like accommodation. Alongside these hardware innovations, the development of custom software tools has become increasingly important to extract the full potential of the data captured by these systems. In particular, custom image analysis algorithms enable precise and tailored quantification of morphological parameters—such as curvature, eccentricity, and shape deformation—beyond the capabilities of standard device software. These developments not only offer novel insights into the functional biomechanics of the eye but also enable age-specific analyses, improve diagnostic precision, and support the development of targeted clinical interventions [18,19,20]. Building on these innovations, the present study introduces a customized conic fitting algorithm applied to radial Scheimpflug images, specifically designed to quantify anterior lens surface geometry under different accommodative demands across the lifespan [21,22,23].

This study aims to describe and apply a novel method for analysing the anterior lens curvature based on high-resolution radial Scheimpflug imaging combined with a custom-developed conic fitting algorithm to quantify lens surface eccentricity in both the horizontal and vertical meridians. The method was used to evaluate morphological variations in response to increasing accommodative demands (0, 1, 3, and 5 dioptres (D)) in a healthy population stratified into five age groups by decade. This approach enables detailed characterization of age-related changes in anterior lens geometry and accommodative response across the lifespan.

## 2. Materials and Methods

### 2.1. Sample Description and Selection

The study adhered to the principles of the Declaration of Helsinki and was approved by the Clinical Research Ethics Committee of Aragón (CEICA) (approval number 23/479).

Participants were eligible for inclusion if they were between 20 and 69 years old, had no binocular or accommodative disorders, and demonstrated a corrected visual acuity of at least 0.8 in the right eye (RE).

Refractive error limits were defined as follows: participants with myopia greater than –5.50 D, hyperopia greater than +2.50 Dor, and astigmatism exceeding 1.50 D were excluded. AA and monocular accommodative facility (MAF) were measured, and values were required to fall within the normal range for each age group, consistent with their expected accommodative capacity. Additional criteria included the absence of ocular or systemic diseases affecting vision, an axial length (AL) between 22 and 26 millimetres (mm), pupil diameter (PD) greater than 4 mm, and an ACD exceeding 2.5 mm. These refractive and biometric thresholds were selected to reduce optical distortion and maintain consistent image quality, thereby enabling accurate modelling of anterior lens curvature and controlling for age-related anatomical variations in anterior segment structures [24,25]. To minimize physiological variability and ensure standardized accommodative responses, participants were instructed to avoid wearing contact lenses and to refrain from consuming caffeine, alcohol, tobacco, or other stimulants for at least two hours prior to each imaging session. These substances can acutely influence pupil diameter, ciliary muscle tone, and accommodative function through their effects on the autonomic nervous system and ocular physiology, potentially confounding measurements of lens curvature and pupil dynamics during accommodation [26,27,28,29].

A total of 104 healthy participants, aged 21 to 62 years, were included in the study, 60 females and 44 males. Only the RE of each participant was included in the analysis to avoid intra-subject correlation and simplify statistical modelling. This approach, commonly adopted in ophthalmic imaging research, reduces redundancy and ensures statistical independence between samples.

The study subjects were divided into five age groups: Group 1 (G1) from 20 to 29 years (*n* = 58), Group 2 (G2) from 30 to 39 years (*n* = 20), Group 3 (G3) from 40 to 49 years (*n* = 12), Group 4 (G4) from 50 to 59 years (*n* = 10), and Group 5 (G5) aged 60 years and older (*n* = 6).

### 2.2. Assessment of Ocular Parameters

AA, measured in D, was assessed for each participant using Donders’ push-up method, with the final value calculated as the average of three consecutive measurements. MAF was evaluated at NV of 40 centimetres (cm) using a ±2.00 D flipper lens, and results were recorded in cycles per minute (cpm).

AL, PD, and ACD were measured using an IOL Master 500 optical biometer (Carl Zeiss Meditec, Oberkochen, Germany). Five measurements were obtained per subject, and values were expressed in mm. Aberrometric measurements were collected using the irx3 Shack–Hartmann aberrometer (Imagine Eyes, Orsay, France), which uses an infrared light source (780 nanometres (nm)) and a microlens sensor array. Measurements were conducted under scotopic lighting conditions and without refractive correction, with participants fixating on a Snellen E target. A fixed pupil size of 4 mm at the corneal plane was used. This assessment determined each participant’s baseline refractive state in Badal space, serving as the reference point (0 D of accommodation) from which accommodative demands were subsequently induced. Thus, the baseline spherical equivalent (M) value was obtained for each subject.

### 2.3. Image Acquisition and Processing

Eight images per eye were acquired using the Galilei G2 Dual Scheimpflug Analyzer corneal topographer (Ziemer Ophthalmic Systems AG, Port, Switzerland). For each eye, one baseline image and three images under induced accommodative demands (1, 3, and 5 D) were recorded, based on each subject’s baseline M obtained from the aberrometer. Additionally, numerical parameters such as PD and the ACD were recorded using the Galilei imaging system.

The Galilei G2 generated ZIP-compressed folders containing raw data for each measurement. These were extracted and processed using a custom-developed software tool, *GalileiConverter 1.0* (University of Zaragoza, Zaragoza, Spain), which preserved the original folder structure and retrieved the 16 axial images of the anterior segment in PNG format. This ensured that the data remained unprocessed by the device’s internal software, maintaining the integrity of the original images.

**Figure 1:**
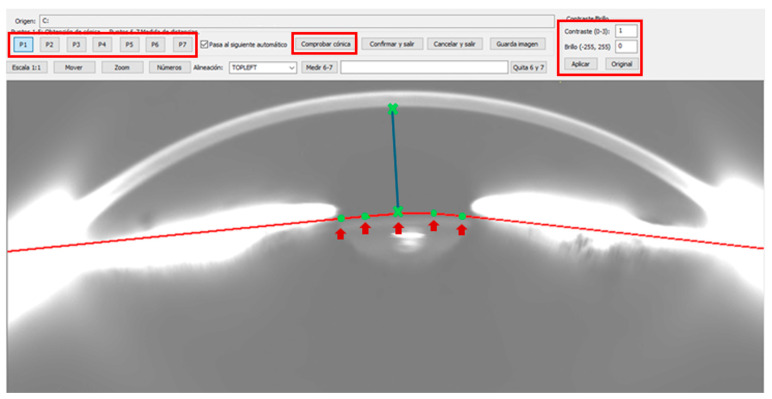
Program interface of *ImgOCT 1.12* software (University of Zaragoza, Zaragoza, Spain) displaying the brightness and contrast settings (boxed in red) corresponding to images from the first Scheimpflug camera. The five points (boxed in red) marked on the anterior lens capsule for the calculation of the conic fit parameters are highlighted in green and with a red arrow. The green crosses correspond to points 6 and 7 (connected by the blue line) used to measure the anterior chamber depth (ACD).

From each set, the 16 frames captured by the first Scheimpflug camera were selected and imported into *ImgOCT 1.12* software (University of Zaragoza, Zaragoza, Spain). Image contrast and brightness were standardized to values of 2 and 100, respectively. Five reference points were manually placed on the anterior lens capsule to extract conic fitting parameters and assess curve changes under different accommodative demands. Manual placement of the five key points in each image was performed by a single trained operator to ensure consistency in identifying the contours of the crystalline lens. To validate the reproducibility of the method, the meridional image number 1 of each subject was analysed and compared with results previously published by Arcas-Carbonell et al. [24], confirming a high concordance between both measurements, thus confirming reproducibility. Subsequently, the analysis was extended to 16 radial cuts per subject, significantly increasing spatial resolution and accuracy in characterizing the anterior lens curvature. Additionally, two additional points—at the corneal apex and lens reflex—were used to measure the ACD, using a scale equivalence of 28 µm per pixel (Figure 1). This scale was determined by comparing known corneal thickness (pachymetry) at the apex with the corresponding pixel count in the images.

The extracted data were automatically exported to a text file and transferred to an Excel database, where lens curvature and ACD values were recorded for all subjects across different accommodative demands (0, 1, 3, and 5 D). Pupillary dilation was not induced in any subject to avoid interfering with the crystalline lens’s natural accommodative capacity. Therefore, the five anterior lens points were placed within the visible portion of the lens capsule, as defined by each subject’s natural pupil aperture. Image acquisition was conducted under the lowest possible ambient lighting conditions to maximize natural pupil dilation and enhance visualization of the anterior lens contour.

The use of these custom-designed software tools enables precise lens curvature measurements with minimal alteration, providing more reliable results compared to traditional methods that rely on enhanced or pre-processed images.

### 2.4. Calculation of Eccentricity, Asphericity, and Shape Factor

The calculation of eccentricity, asphericity (*Q*), and shape factor (*SF*) were calculated by fitting the anterior lens curvature using the general conic Equation (1). This method, widely used in the characterization of optical surfaces, has been previously described in detail in prior studies [24] (Appendix A).

(1) $${ax}^{2}+2hxy+{by}^{2}+2gx+2fy+n=0$$

These dimensionless parameters allow for the evaluation of curvature variations in the anterior lens capsule in response to increasing accommodative demand, providing a precise characterization of the geometry of the structure.

### 2.5. Reconstruction of the Cuts into a Single Figure

The study sample consisted of 104 subjects, with up to 16 radial cuts acquired per subject, resulting in an initial dataset of 1664 images. All images demonstrated very high quality, allowing for detailed visualization of the anterior segment structures.

To optimize the interpretation and organization of the data, a grouping strategy was implemented by dividing the cuts into two anatomically coherent sectors.

**Figure 2:**
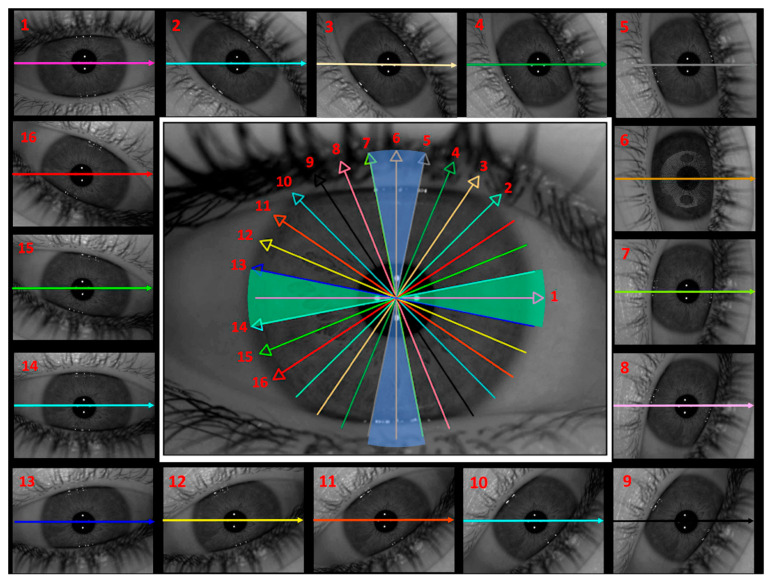
Spatial distribution of the 16 cuts obtained with the Scheimpflug system on the ocular surface. Each cut is represented in a different colour to facilitate identification, corresponding to the rotated eye image provided by the Scheimpflug camera (Ziemer Ophthalmic Systems AG, Port, Switzerland). Additionally, the cut numbers, shown in red, indicate their specific position, while the coloured arrows point to the corresponding direction of each cut. This representation allows for a better understanding of the system’s scanning trajectory and the arrangement of the cuts in the analysis. To enhance interpretation, the cuts have been grouped into two sectors, shown in the central image, each represented by a different colour. The horizontal sector, in green, corresponds to the horizontal meridian, while the vertical sector, in blue, aligns with the vertical meridian. Each meridian encompasses an angular range of 22.5°, allowing for a focused analysis of curvature variations along these principal orientations.

Figure 2 illustrates the spatial distribution of the 16 radial cuts across the eye, reflecting the rotational scanning pattern of the dual-camera Scheimpflug system. The figure also shows the two defined sectors aligned with the main horizontal and vertical meridians, which have been logically and systematically organized to facilitate analysis. The segmentation was performed as follows: the horizontal sector (green) included cuts 1, 13, and 14, representing the horizontal meridian, while cuts 8 to 12 served as transitional cuts. The vertical sector (blue) included cuts 5, 6, and 7, corresponding to the vertical meridian, with cuts 2 to 4 and 15 to 16 serving as transitional cuts. Each meridian encompassed an angular range of 22.5°, enabling a symmetrical and direction-specific analysis of anterior lens curvature.

This systematic organization improved the topographic representation of the cuts, facilitating data interpretation and allowing for more precise comparisons across participants.

### 2.6. Statistical Analysis

All data collected in this study were initially organized and exported in Excel spreadsheets and subsequently processed using a custom Python-based statistical pipeline developed in Google Colab (Google LLC), running Python version 3.11.13. Descriptive statistics, including means, standard deviations (SDs), and ranges (minimum and maximum values), were computed for all quantitative variables.

To examine associations between age and accommodative function, Pearson correlation analyses were conducted between age and both AA and MAF. For each correlation, the Pearson correlation coefficient (r), associated *p*-value, and linear regression equation were reported to quantify the strength and direction of the associations.

Changes in PD as a function of accommodative stimulus and age were assessed using the Friedman test for repeated measures, applied both to the total group (TG) and to each age-stratified subgroup. To account for multiple comparisons across six possible pairwise contrasts, Bonferroni correction was applied, setting a more stringent significance threshold of *p* < 0.0083.

Relative changes in lens eccentricity were analysed in relation to accommodative effort and age using a combination of non-parametric tests. The Wilcoxon signed-rank test was used to compare eccentricity between horizontal and vertical meridians at each accommodative demand level (0, 1, 3, and 5 D) within the TG and all subgroups. The Kruskal–Wallis test assessed differences in eccentricity across age groups for each accommodative level and meridian. Additionally, the Friedman test was employed to evaluate within-group variations in eccentricity across the four levels of accommodation. Post hoc pairwise comparisons following the Friedman test were adjusted using Bonferroni correction (*p* < 0.0083). For all other analyses where multiple testing correction was not applied, statistical significance was set at the conventional level of *p* < 0.05.

Lastly, variations in ACD across accommodative demand and age groups were analysed similarly using the Friedman test with Bonferroni-adjusted post hoc comparisons, mirroring the analytical approach applied to PD.

## 3. Results

A total of 104 REs from 104 healthy participants were included in this study, comprising 44 men (41.18%) and 60 women (58.82%), with a mean age of 34.08 ± 12.04 years (age range: 21 to 62 years). In the TG, the mean AL was 24.27 ± 1.11 mm, the mean ACD was 3.52 ± 0.37 mm, the mean PD was 6.52 ± 0.98 mm, and the mean SE was –2.78 ± 2.45 D (Table 1, TG).

The participants were divided into five age groups: G1 from 20 to 29 years (*n* = 58), G2 from 30 to 39 years (*n* = 20), G3 from 40 to 49 years (*n* = 12), G4 from 50 to 59 years (*n* = 10), and G5 aged 60 years and older (*n* = 6).

According to the visual function, the TG showed a mean AA of 13.16 ± 13.02 D and a mean MAF of 8.49 ± 6.71 cpm (Table 1, TG). As shown in Table 1, both AA and MAF exhibited a decreasing trend with advancing age.

The real accommodative ability of the participants was also evaluated under increasing accommodative demands (0, 1, 3, and 5 D).

The PD and ACD, measured by the optical biometer, also showed an age-related decline. Specifically, the younger groups G1 (6.79 ± 0.90 mm and 3.62 ± 0.37 mm, respectively) and G2 (6.30 ± 1.09 mm and 3.67 ± 0.23 mm, respectively) exhibited larger mean PDs and ACDs, whereas the older groups G3 (6.11 ± 1.09 mm and 3.51 ± 0.40 mm, respectively), G4 (6.07 ± 0.99 mm and 3.15 ± 0.44 mm, respectively), and G5 (5.89 ± 0.57 mm and 2.78 ± 0.10 mm, respectively) showed progressively smaller values.

### 3.1. Accommodative Function Correlations with Age

**Figure 3:**
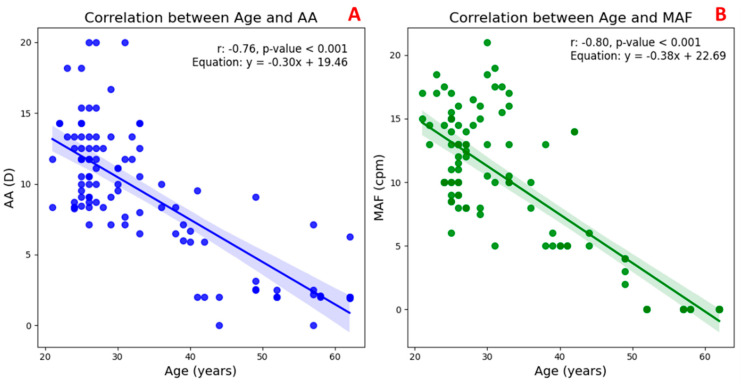
Scatter plots with regression lines and their equations, as well as the correlation coefficients and statistical significance between TG participants’ age (20–65 years) and two measures of accommodative function: amplitude of accommodation (AA, blue line (**A**)) and monocular accommodative facility (MAF, green line (**B**)).

Figure 3 illustrates the correlation between the age and two key indicators of accommodative function in the TG.

In Figure 3A, a significant negative correlation can be observed between age and AA (*r* = –0.76, *p* < 0.001), indicating a marked decline in the eye’s ability to increase optical power with advancing age. Similarly, Figure 3B shows a strong inverse relationship between age and MAF (*r* = –0.80, *p* < 0.001), reflecting a reduction in the speed and flexibility of the accommodative response over time. These findings confirm that both the magnitude and efficiency of accommodation diminish progressively with age, consistent with the physiological changes associated with presbyopia.

### 3.2. Pupil Diameter Variations with Accommodation and Age

**Figure 4:**
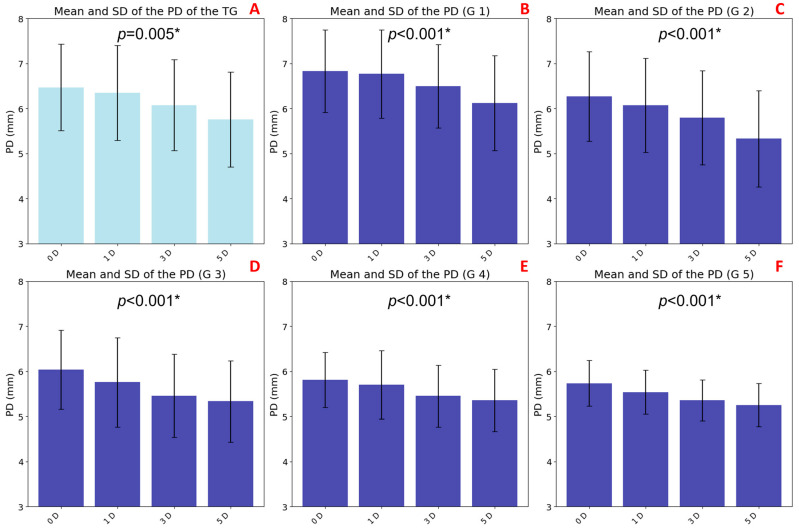
Bar graphs representing the mean pupil diameter (PD) values in response to accommodative stimuli of 0, 1, 3, and 5 D, with error bars indicating standard deviations. Graph (**A**) shows data from the total group (TG), while graphs (**B**–**F**) correspond to age groups G1 to G5, respectively. Statistically significant differences across accommodative levels, as determined by the Friedman test (*p* < 0.0083 with Bonferroni correction), are indicated with an asterisk (*).

A statistically significant decrease in PD was observed with increasing levels of accommodative demand (*p* = 0.005), indicating a consistent and robust pupillary response across the TG sample. This reduction, known as accommodative miosis, followed a progressive trend, with the most notable change occurring between the baseline (0 D with 6.47 ± 0.96 mm) and moderate accommodation (5 D with 5.75 ± 1.05 mm) (Figure 4A).

Age-stratified analysis also revealed significant differences (*p* < 0.001) in all groups in baseline PD values with magnitude of accommodative miosis. Younger participants—G1 (6.83 ± 0.91 mm), G2 (6.27 ± 0.99 mm), and G3 (6.04 ± 0.87 mm)—exhibited larger baseline PDs and a more pronounced pupillary constriction in response to increasing accommodative stimuli (Figure 4B, 4C, and 4D, respectively). In contrast, older groups—G4 (5.81 ± 0.61 mm) and G5 (5.74 ± 0.51 mm)—showed smaller baseline PDs and a markedly attenuated pupillary response, indicating an age-related decline in both pupillary dynamics and accommodative responsiveness (Figure 4E, and 4F, respectively). 

### 3.3. Relative Lens Eccentricity Results According to Accommodative Effort and Age

**Figure 5:**
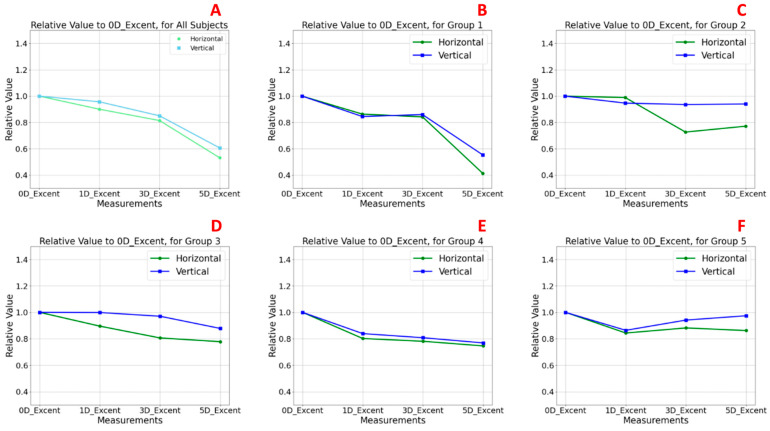
Changes in relative anterior lens eccentricity during accommodation. Graph (**A**) represents the total group (TG), while panels (**B**–**F**) show subgroup-specific analyses by age (G1–G5). The X-axis indicates the accommodative stimulus levels (0, 1, 3, and 5 D), and the Y-axis displays relative eccentricity values, normalized to the baseline condition (0 D = 1.0). The green lines represent the horizontal meridian, and the blue lines represent the vertical meridian. Values above 1.0 reflect increased anterior lens eccentricity compared to baseline, corresponding to a relative flattening of the lens surface, whereas values below 1.0 indicate decreased eccentricity, consistent with an increase in anterior surface curvature during accommodation.

Figure 5 illustrates the relative changes in anterior lens surface eccentricity in response to increasing accommodative demands, normalized to baseline values at 0 D. Figure 5A presents the analysis of the TG, while Figure 5B–F correspond to the age-stratified subgroups (G1–G5). Baseline values at 0 D were normalized to 1.0, and eccentricity values at 1, 3, and 5 D were expressed relative to this reference. Values above 1.0 indicate an increase in eccentricity (i.e., a flatter curvature), whereas values below 1.0 suggest a reduction in eccentricity, implying a more curved anterior lens surface.

In the TG (Figure 5A), both the horizontal and vertical sectors exhibited a statistically consistent pattern of decreasing eccentricity with increasing accommodative demand, with this effect being particularly evident at 5 D. At baseline, eccentricity relative values were 1.0 in both meridians, decreasing to 0.531 (horizontal) and 0.606 (vertical) at 5 D. No statistically significant differences were observed between meridians at any accommodative level (Table 2). These findings indicate a significant increase in anterior lens surface curvature during accommodation, consistent with the classical Helmholtz model [30,31]. The effect was slightly more pronounced in the vertical meridian across all stimulus levels.

In the youngest subgroup (G1, Figure 5B), this pattern was most pronounced, with clear reductions in eccentricity at higher accommodative levels, particularly at 5 D (horizontal sector eccentricity: 0.414; vertical sector eccentricity: 0.491) compared to the baseline at 0 D (horizontal and vertical sector eccentricity: 1.0), although no statistically significant differences were observed in any case between meridians among accommodative levels (Table 2). These findings confirm an effective anterior lens surface remodelling mechanism during accommodation, with slightly greater curvature changes in the vertical sector.

In G2 (Figure 5C), a similar but less pronounced pattern to that observed in G1 was found. Although eccentricity continued to decrease with increasing accommodative demand, the changes were less moderate, and a slight peak at 3 D in the horizontal meridian suggested a transitional phase in accommodative capacity. Eccentricity values in the horizontal sector were 0.989 at 1 D, 0.726 at 3 D, and 0.772 at 5 D. In the vertical sector, the values were 0.946 at 1 D, 0.936 at 3 D, and 0.939 at 5 D. As in G1, no statistically significant differences were found between meridians among accommodative levels (Table 2).

In contrast, groups G3 (Figure 5D), G4 (Figure 5E), and G5 (Figure 5F) showed no clear trend in eccentricity changes. Relative values remained close to baseline across the different levels of accommodative demand, indicating a reduced capacity for anterior lens surface reshaping. In G3, slightly higher relative eccentricity values were observed, with horizontal sector values of 0.895 at 1 D, 0.806 at 3 D, and 0.777 at 5 D and vertical sector values of 0.998 at 1 D, 0.971 at 3 D, and 0.878 at 5 D. In G4, relative eccentricity values in the horizontal sector were 0.802 at 1 D, 0.781 at 3 D, and 0.746 at 5 D, while in the vertical sector they were 0.839 at 1 D, 0.808 at 3 D, and 0.768 at 5 D. These results are consistent with the age-related decline in accommodative function with minimal curvature changes, particularly along the horizontal meridian. G5, the oldest group, displayed the most stable eccentricity values (horizontal sector: 0.843 at 1 D, 0.882 at 3 D, and 0.862 at 5 D; vertical sector: 0.864 at 1 D, 0.942 at 3 D, and 0.974 at 5 D), suggesting a nearly static anterior lens profile during accommodative attempts. Once again, no statistically significant differences were observed in any case between sectors across accommodative levels (Table 2).

Notably, across the TG and all age groups, the vertical meridian consistently showed slightly higher eccentricity values than the horizontal, with the highest values occurring at baseline (0 D).

#### Statistical Comparison of Horizontal and Vertical Sectors

To assess potential differences in relative eccentricity between the horizontal and vertical meridians, Wilcoxon signed-rank tests were performed for the TG and each age subgroup across all accommodative levels (0, 1, 3, and 5 D). The results, summarized in Table 2, revealed no statistically significant differences between meridians under any condition (*p* > 0.05). However, though non-significant, the trend was consistently observed in which the vertical meridian exhibited slightly higher relative eccentricity values than the horizontal meridian across all groups. This pattern suggests a subtle tendency for the horizontal anterior lens surface to undergo greater steepening (i.e., increased curvature) during accommodation. Furthermore, the highest eccentricity values were consistently recorded at baseline (0 D), indicating that the lens adopts its flattest configuration in the absence of accommodative effort.

To investigate potential age-related differences in relative lens eccentricity, separate analyses were conducted for the horizontal and vertical sectors across the five age groups (G1–G5). For each level of accommodative demand (0, 1, 3, and 5 D), Kruskal–Wallis tests were employed to assess whether significant differences existed among the groups. The results, summarized in Table 3, revealed no statistically significant differences between age groups at any accommodative level (*p* > 0.05). Consequently, no post hoc pairwise comparisons (e.g., Mann–Whitney tests) were performed, and no Bonferroni correction for multiple comparisons was applied. However, some non-significant trends were observed in the data. Specifically, eccentricity values tended to vary more in the younger groups (G1 and G2) compared to the older groups (G3–G5), where eccentricity remained relatively flat despite increasing accommodative demand. Although these findings do not reach statistical significance, they may reflect age-related morphological changes in anterior lens geometry, being more evident when subjects still retain accommodative capacity and diminishing as this capacity declines.

To evaluate differences in relative lens eccentricity between the induced accommodative levels (0, 1, 3, and 5 D) within the TG and within each age group for each meridian (horizontal and vertical), non-parametric Friedman tests for related samples were conducted. Post hoc pairwise comparisons were adjusted using Bonferroni correction to account for the six possible comparisons, setting the significance threshold at *p* = 0.0083 (Table 4). Statistically significant differences were found only in the horizontal meridian of the TG (*p* = 0.002), indicating a significant increase in curvature (i.e., decrease in eccentricity) in response to higher accommodative demands. In contrast, no statistically significant differences were observed across accommodative levels in the vertical meridian or within the age-stratified subgroups (*p* > 0.0083). These findings align with previous observations suggesting limited but directional changes in anterior lens shape during accommodation, with the horizontal meridian showing a greater dynamic response.

### 3.4. ACD Variations with Accommodation and Age

**Figure 6:**
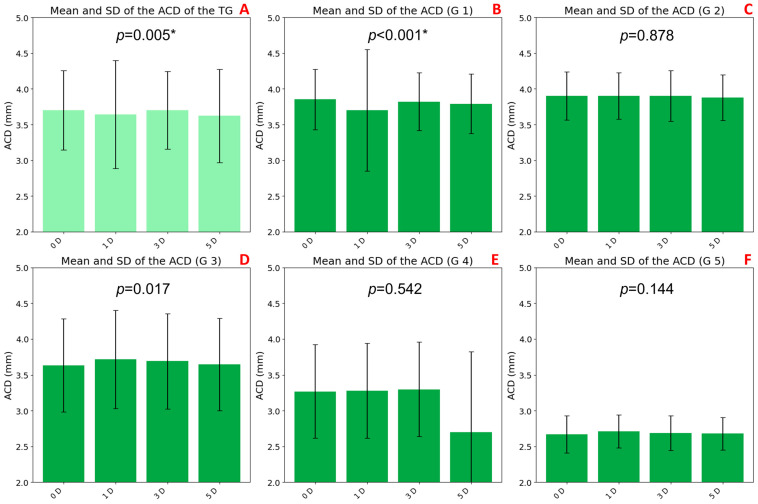
Bar graphs representing mean anterior chamber depth (ACD) values under different accommodative stimuli (0, 1, 3, and 5 D), with standard deviations shown as error bars. Graph (**A**) shows data from the total group (TG), while graphs (**B**–**F**) correspond to age groups G1 to G5, respectively. Statistically significant differences across accommodative levels, as determined by the Friedman test (*p* < 0.0083 with Bonferroni correction), are indicated with an asterisk (*).

A statistically significant decrease in ACD was observed with increasing accommodative demand in the TG (*p* = 0.005; Figure 6A). This reduction was more pronounced at 5 D (3.62 ± 0.65 mm) compared to the baseline at 0 D (3.70 ± 0.56 mm).

Age-stratified analysis revealed significant changes only in group G1 (*p* < 0.001; Figure 6B), where ACD progressively decreased from 0 D (3.95 ± 0.42 mm) to 3 D (3.82 ± 0.40 mm) and 5 D (3.79 ± 0.42 mm). No significant differences were found in groups G2 through G5.

Baseline ACD values also varied across age groups. Older participants (G4: 3.27 ± 0.65 mm and G5: 2.67 ± 0.26 mm) had shallower anterior chambers, while younger groups showed higher baseline ACD values (G1: 3.95 ± 0.42 mm, G2: 3.90 ± 0.34 mm, and G3: 3.63 ± 0.65 mm). These trends were confirmed by biometric measurements under non-accommodative conditions, as presented in Table 1 (G1: 3.62 ± 0.37mm, G2: 3.67 ± 0.23 mm, G3: 3.51 ± 0.40 mm, G4: 3.15 ± 0.44 mm, and G5: 2.78 ± 0.10 mm).

## 4. Discussion

This study introduced and applied a novel method to evaluate age-related morphological changes in the anterior surface of the crystalline lens in response to increasing accommodative demands. Using high-resolution radial Scheimpflug imaging combined with a custom conic fitting algorithm, lens surface eccentricity was quantified in both horizontal and vertical meridians across five age groups. This approach enabled the detection of subtle yet measurable geometric adaptations of the lens, offering valuable insights into the biomechanical behaviour of the accommodative system throughout the lifespan. These changes may reflect compensatory mechanisms of the visual system, such as age-related adaptive responses, including altered lens biomechanics, reduced ciliary muscle contraction efficiency, and shifts in zonular tension. These adaptations attempt to preserve accommodation despite declining elasticity and the progressive reduction in accommodative capacity associated with aging.

Overall, the results indicate that in younger individuals, particularly those in group G1, accommodation is associated with a significant increase in anterior lens curvature, reflected by a decrease in eccentricity more pronounced in the vertical meridian. In contrast, older subjects exhibit minimal or no curvature change, consistent with presbyopic alterations in lens biomechanics [25].

Both intrinsic material-property modifications of the lens and geometric alterations contribute to accommodative decline; however, it is the age-related stiffening of the lenticular substance that exerts the most substantial impact. Intriguingly, these changes do not alter the distribution of zonular tension but rather modify the angles at which the zonular fibres insert into the lens surface [32].

It is also important to highlight, as previously described by Grewal et al. [33], that the optical design of Scheimpflug cameras, which relies on the tilting of the image plane relative to the lens and object planes, provides superior focus sharpness across the captured cross-section compared to conventional parallel-plane imaging systems. This optical advantage enhances the accuracy of crystalline lens curvature assessment across different meridians, increasing confidence in the structural measurements reported in this study.

An important aspect highlighted in this study is the behaviour of PD in response to increasing accommodative demand. Statistically significant differences (*p* < 0.05 in all cases) were observed both in the TG and across all age-stratified subgroups, supporting the consistency and robustness of the findings. A progressive reduction in PD, indicative of accommodative miosis, was evident as accommodative demand increased, particularly between 0 and 3 D. This trend, observed consistently across the entire sample, reflects the well-established physiological coupling between accommodation and pupillary constriction (e.g., via the near triad reflex) [34].

Age group analysis revealed significant differences in both baseline PD values and the magnitude of miosis. Younger individuals (G1, G2, and G3) exhibited larger resting PDs and a more pronounced relative constriction in response to accommodative effort, suggesting a more effective pupillary reflex. In contrast, older participants (G4 and G5) showed smaller baseline PDs and an attenuated pupillary response, consistent with previous reports describing age-related decline in pupillary reactivity [35,36].

The attenuated miosis observed in older groups may be attributed to reduced parasympathetic tone [37,38] or structural changes in the iris musculature [39], both of which could compromise the effectiveness of the accommodative response. These findings align with age-related physiological changes described by Zapata-Díaz et al. [40], who developed an age-dependent eye model based on in vivo measurements. Their model highlights a progressive decline in AA and pupillary dynamics with age, reinforcing the current observations of reduced pupillary reactivity and accommodative efficiency in older individuals [40].

One of the most noteworthy findings of this study was the behaviour of ACD in response to increasing accommodative stimuli. Statistically significant differences were observed in the TG (*p* = 0.05) and in subgroup G1 (*p* < 0.01), indicating that the observed changes in ACD are unlikely to be due to chance. Overall, ACD progressively decreases with increasing accommodative demand, reflecting the anterior displacement of the crystalline lens during accommodation, particularly at higher stimulus levels (3 and 5 D). When stratified by age, distinct patterns emerged. Younger groups (G1, G2, and G3) exhibited greater variability and a more dynamic ACD response, suggesting a more effective accommodative mechanism. Notably, G1 showed highly significant changes (*p* < 0.001), reinforcing this interpretation. In contrast, older groups (G4 and G5) showed more limited changes in ACD and shorter chambers in the unaccommodated state, consistent with age-related reductions in lens elasticity and accommodative capacity [25]. A limitation of the study is the uneven gender distribution across age groups, particularly in the older cohorts (G4 and G5). While no statistically significant sex-related differences were found in any of the measured variables, this imbalance may obscure subtle dimorphic trends in lens biomechanics and aging. Future studies with balanced sex representation are recommended to confirm these results. Furthermore, the non-significant findings observed in the oldest age group (G5) may be attributed to the small sample size (*n* = 6). This limits the statistical power to detect subtle age-related changes. New studies should aim for larger and more balanced recruitment in older populations to validate these observations. These findings align with previous studies reporting age-related declines in the biomechanical flexibility of the lens and zonular apparatus [41], which likely contribute to a diminished accommodative amplitude. Moreover, these findings are consistent with previous research reporting both the presence and absence of sex-related differences, depending on the studied population and the measurement methods used [42,43,44]. For instance, some studies have shown that males tend to have a greater anterior chamber depth and axial length [42,44], whereas other studies based on objective accommodative measurements found no significant differences between sexes [43]. This variability highlights the importance of conducting future research with larger, more representative, and sex-balanced samples to better elucidate the role of sex in ocular biometric changes across the lifespan.

Importantly, these results are consistent with those reported by Arcas et al. [24], who observed similar age-related trends using a single meridional image per subject. The present study strengthens those findings by increasing the sampling resolution to 16 radial cuts per subject, thereby enhancing the robustness and reproducibility of the measurements and supporting the validity of the previously reported observations [24].

To reduce inter-observer variability and improve reproducibility, an automated system based on AI-assisted segmentation is currently under development, which will be implemented and evaluated in future studies.

Many existing methods for measuring and predicting the properties of crystalline lenses rely heavily on the analysis of ex vivo or in vitro lenses [45,46,47]. These approaches, while valuable, often do not capture the natural physiological conditions of the lens in a living eye, which can affect accuracy and applicability. In contrast, our study distinguishes itself by employing in vivo data, allowing for a more realistic and clinically relevant assessment of the crystalline lens. This in vivo approach enables the capture of dynamic changes and preserves the native biomechanical environment, thereby providing insights that are closer to actual physiological conditions compared to traditional ex vivo or in vitro methodologies.

Despite supporting previous findings, some methodological choices may have limited measurement accuracy. Key factors like pupil size, eye movements, and optical access under natural conditions are discussed below as potential sources of variability.

First, a key methodological decision in this study was to avoid the use of mydriatic agents, thereby preserving the natural accommodative response of the crystalline lens and allowing assessment under conditions that closely reflect normal visual function [48]. While this strategy enables a more realistic evaluation of accommodation, it also involves certain trade-offs. The lack of pharmacological pupil dilation may reduce the quality and accuracy of peripheral measurements of the anterior lens surface [49,50], particularly in older subjects, in whom PD is significantly reduced [36,51]. This age-related decline in pupil size is consistent with previously described physiological changes in the iris musculature and in the autonomic control of pupillary function [52]. In our study, reduced pupil area hindered the precise identification of the anterior lens margins, increasing variability in curvature measurements at the periphery, especially when the available area for analysis was limited.

Although cycloplegic agents were not used, it is important to acknowledge that pupil dilation, even without inducing accommodative paralysis, can indirectly influence the accommodative response by disrupting the balance of the near triad (accommodation, convergence, and miosis). This phenomenon has been previously reported and represents a potential confounding factor when comparing results across studies with differing pharmacological protocols [34,50].

Another important limitation of not using mydriasis is the restricted ability to assess deeper lens structures, such as total lens thickness. Future investigations should consider incorporating imaging modalities with greater tissue penetration, such as SS-OCT [53,54] or high-frequency ultrasound [55]. These technologies could complement Scheimpflug-derived data, particularly under dilated conditions, enabling access to additional parameters without substantially interfering with accommodative physiology [56].

It is also worth noting that, while these imaging techniques are non-invasive and safe, they require stable fixation and are susceptible to motion artifacts. In the case of Scheimpflug, approximately 25 to 50 scans are acquired over a period of 2 s as the camera rotates around the eye. A notable advantage of the device used in our study is its dual-camera system, which enables signal averaging and reduces decentration errors caused by involuntary eye movements during acquisition [57,58].

Despite these limitations, the present study successfully meets its primary objective: to validate a novel methodological approach using a custom conic fitting algorithm for quantifying anterior lens surface geometry under varying accommodative demands. The results provide a robust foundation for refining and applying this protocol in future research.

Unlike conventional approaches relying on single-plane curvature measurements or device-embedded algorithms [59], the conic fitting methodology presented in this study offers several advantages. First, it enables a high spatial resolution by analysing 16 radial cuts, providing a more comprehensive curvature profile of the anterior lens surface [60]. Second, it allows direct quantification of eccentricity in both the horizontal and vertical meridians, facilitating the detection of subtle geometric changes related to accommodation. Moreover, this method reduces dependence on proprietary interpolation algorithms embedded in commercial devices, which often lack transparency and reproducibility [61]. Finally, the use of raw Scheimpflug images allows for full methodological customization and reproducibility [62]. These features enhance the precision and interpretability of anterior lens curvature analysis, particularly in studies of accommodative biomechanics.

Building upon these findings, the novel conic fitting method presented here not only facilitates detailed morpho-functional assessment of the anterior crystalline lens surface but also holds important clinical and research potential. Its ability to detect subtle age-related and accommodative biomechanical changes supports improved diagnosis and monitoring of presbyopia progression. Moreover, the method can be applied for evaluating the performance of accommodative intraocular lenses in pre- and post-operative settings. Given its non-invasive, high-resolution, and in vivo approach, this technique is well suited for longitudinal clinical studies, providing valuable insights into lens dynamics and accommodation mechanisms over time.

## 5. Conclusions

This study validates a novel method for evaluating anterior lens curvature using Scheimpflug imaging and conic analysis, revealing age-related declines in accommodation. Both PD and ACD decreased with accommodative effort and age. Younger subjects showed significant lens curvature changes, with a non-significant trend suggesting a slightly greater reduction in the vertical meridian. In contrast, older groups exhibited minimal variation, reflecting presbyopic biomechanical limitations. These findings deepen understanding of accommodative biomechanics and support the method’s potential for future applications.

## Figures and Tables

**Table 1:** Characteristics of the sample, including the number of participants (*n*), gender distribution with counts of males and females (M/F), mean and standard deviation (± SD) of pupil diameter (PD) in millimetres (mm), spherical equivalent (SE) in dioptres (D), accommodative amplitude (AA) in D, monocular accommodative facility (MAF) in cycles per minute (cpm), axial length (AL) in mm, and anterior chamber depth (ACD) in mm of the right eye, presented for the total group (TG) as well as for each age group.

	n	Age	Gender (M/F)	PD (mm)	SE (D)	AA (D)	MAF (cpm)	AL (mm)	ACD (mm)
TG	104	34.27 ± 12.04	44/60	6.52 ± 0.98	−2.42 ± 2.09	8.96 ± 4.94	9.07 ± 6.19	24.06 ± 2.40	3.53 ± 0.37
G1	58	25.68 ± 1.98	18/40	6.79 ± 0.90	−2.20 ± 2.16	11.61 ± 3.37	12.15 ± 4.14	23.96 ± 0.88	3.62 ± 0.37
G2	20	33.60 ± 2.98	12/8	6.30 ± 1.09	−3.35 ± 2.02	9.72 ± 4.41	9.76 ± 6.73	24.56 ± 1.11	3.67 ± 0.23
G3	12	44.17 ± 3.79	4/8	6.11 ± 1.09	−3.58 ± 1.81	4.27 ± 3.06	6.00 ± 4.15	24.65 ± 0.90	3.51 ± 0.40
G4	10	55.20 ± 2.78	8/2	6.07 ± 0.99	−1.30 ± 1.03	2.44 ± 1.80	0	21.40 ± 4.62	3.15 ± 0.44
G5	6	61.33 ± 0.58	2/4	5.89 ± 0.57	−1.00 ± 1.51	1.98 ± 0.03	0	23.91 ± 1.91	2.78 ± 0.10

**Table 2:** *p*-values obtained from the Wilcoxon signed-rank test comparing relative lens eccentricity between the horizontal and vertical sectors at each level of accommodative demand (0, 1, 3, and 5 D) for the total sample (TG) and for each age group (G1 to G5).

Accommodative Demand	Horizontal vs. Vertical Eccentricity (*p*-Values)			
	0 D	1 D	3 D	5 D
TG	0.419	0.512	0.739	0.845
G1	0.651	0.632	0.712	0.975
G2	0.557	0.734	0.375	0.844
G3	1.000	0.625	0.125	0.500
G4	0.625	0.625	0.500	0.750
G5	0.750	1.000	0.750	1.000

**Table 3:** The *p*-values obtained from Kruskal–Walls test comparing relative lens eccentricity across the five age groups (G1–G5) for each accommodative demand level (0, 1, 3, and 5 D), separately analysed for horizontal and vertical sectors.

Sector	G1 vs. G2 vs. G3 vs. G4 vs. G5 Eccentricity (*p*-Values)			
	0 D	1 D	3D	5D
Horizontal	0.609	0.386	0.098	0.805
Vertical	0.377	0.119	0.288	0.169

**Table 4:** *p*-values obtained from Friedman related test comparing relative lens eccentricity between the induced accommodations (0, 1, 3, and 5 D) within the total group (TG) and within each of the five groups (G1–G5) for horizontal and vertical sectors separately. Post hoc pairwise comparisons were performed with Bonferroni correction for six possible comparisons, setting significance at *p* = 0.0083, marked with an asterisk (*).

0 D vs. 1 D vs. 3D vs. 5D Eccentricity (*p*-Values)		
Group	Horizontal	Vertical
TG	0.002 *	0.028
G1	0.019	0.104
G2	0.160	0.327
G3	0.782	0.241
G4	0.719	0.392
G5	0.308	0.801

## Data Availability

The data presented in this study are available upon request from the corresponding author.

## Appendix A

The calculation of (*e*, *Q*, *SF*) for both retinal and lens curvature was based on fitting the curve according to the general conic Equation (A1):

(A1) $$ax^{2}+2hxy+by^{2}+2gx+2fy+n=0$$

The intersection of a plane with a cone, when it is not passing through the vertex, results in four distinct types of curves: hyperbolas, parabolas, ellipses, and circles [63,64]. As the *e* value of an ellipse approaches 1, the shape of the conic section gradually flattens. When *e* = 1, the resulting figure becomes a parabola, and with *e* = 0, a perfect circle is obtained. Therefore, as *e* increases, the ellipse tends to flatten. When *e* > 1, the conic section becomes a hyperbola [65].

The data for each subject measured in the images by the ImgOCT 1.12 program are governed by a series of coordinates that vary based on the arrangement of each point. These coordinates are used to calculate the parameter *e*, Equation (A2):

(A2) $$e^{2}=\;2\sqrt{{\left(a-b\right)}^{2}+4h^{2}}/(\sigma \left(a+b\right)+\sqrt{{\left(a-b\right)}^{2}+4h^{2}}\;)$$

To determine the sign (σ) of e, the program uses the following formula Equation (A3):

(A3) $$\sigma =sign\;(nh^{2}-abn+af^{2}-2hfg+bg^{2})$$

The Q value describes the degree of variation in curvature of a surface from its center to its periphery Equation (A4). *Q* = 0 indicates a spherical surface, with no difference between the central and peripheral radii. If *Q* < 0, this signifies a prolate profile with progressive flattening towards the periphery, meaning the center is more curved than the periphery. Conversely, if *Q* > 0, this denotes an oblate profile, where the periphery has greater curvature than the center of the surface [66,67].

(A4) $$Q=-e^{2}$$

Finally, the SF combines (*e*, *Q*) to characterize the precise shape of the surface Equation (A5):

(A5) SF=1−Q

These parameters used to assess the curvature of the lens are dimensionless, without measurement units. In this work, these parameters were applied to examine the changes occurring in the surface of the anterior lens capsule as the accommodative demand increases.
